# Transcriptional Regulation in Rocket Leaves as Affected by Salinity

**DOI:** 10.3390/plants9010020

**Published:** 2019-12-23

**Authors:** Giulia Franzoni, Giacomo Cocetta, Alice Trivellini, Antonio Ferrante

**Affiliations:** 1 Department of Agricultural and Environmental Sciences, Università degli Studi di Milano, via Celoria 2, 20133 Milano, Italy; antonio.ferrante@unimi.it; 2 Institute of Life Sciences, Scuola Superiore Sant’Anna, Pz. Martiri della Libertà 33, 56127 Pisa, Italy

**Keywords:** borage extract, *Diplotaxis tenuifolia* L., salt stress, transcription factors

## Abstract

Salinity is one of the major abiotic stress causing yield losses and decreasing product quality. The beneficial effects of biostimulant products to enhance plant tolerance to abiotic stresses have been reported in several crops, but their mode of action is poorly understood. This work aims to better understand the effect of salt stress on wild rocket treated with a borage extract. The expression of some of the transcription factors (TFs) typically involved in salt stress response was studied within a 24 h period. Physiological parameters such as chlorophyll, chlorophyll *a* fluorescence, carotenoids, phenols, and anthocyanin were analyzed. Results obtained showed that salt stress induced a general increase in the expression levels of almost all TFs studied, whereas the treatment with the plant-base extract only induced an increase at specific time points. Moreover, the approach adopted allowed indagating the change in gene expression during time. Different pathways such as sugars metabolism, cuticular wax biosynthesis, and brassinosteroids signaling took part in plant responses.

## 1. Introduction

Salinity stress is a major abiotic stress affecting plant growth, quality, and productivity. More than 30% of the irrigated land of the world is affected by salinization and this number was projected to increase considering the climate change scenario and the environmental pollution [1,2]. Therefore, the study of the physiological and molecular mechanisms of tolerance to salt stress is crucial to obtain crops able to survive without loss of productivity under stressful conditions.

It is known that salt stress impairs plants ‘performance for different reasons: it causes hyperosmotic stress, ion imbalance and, as consequence, oxidative damage. In the short term (after minutes, hours, and the first day of stress), salt stress is perceived by the roots as osmotic stress caused by the reduced ability of the plants to take up water. In the long term (days, weeks, or years), plant growth is limited by the ion toxicity and nutrient imbalance occurring in the cytoplasm due to the accumulation of salt and the competitive uptake mechanisms with other ions. The first phase, when the salt has not penetrated in plant tissues, is also known as the water-deficit effect of salinity and the cellular and metabolic processes involved are in common with drought stress. The second phase is the salt-specific effect and it is due to the excess of ions inside the plant [3].

Several metabolic processes such as photosynthesis [4,5], respiration [6], phytohormones regulation [7], and protein biosynthesis can be altered by salinity. The effects of the stress on plants vary depending on different factors, such as the level of salt concentration, the duration of the exposure, the phenological stage, the interaction with the environmental conditions, and the ability of a species or cultivar to grow in saline condition. According to this ability, plants are generally divided into glycophytes and halophytes. Glycophytes growth is inhibited by concentrations of NaCl around 100–200 mM, whereas halophytes can survive at higher concentrations of NaCl, typically around 300–500 mM [8]. The threshold value used as criteria to define if a plant belongs to glycophytes or halophytes changed during time and according to different authors. For instance, Flowers et al. [9] initially proposed a value of 300 mM and then 200 mM, whereas other authors referred to a lower threshold about 85 mM [10]. This situation has led to a confusion in the number of the species belonging to one or the other categories.

Wild rocket (*Diplotaxis tenuifolia* L.) is a perennial plant grown in the Mediterranean basin present in coastal areas that has been recently classified as salt tolerant with potential as vegetable crops for saline agriculture. De Vos et al. [11] investigated the responses of this species under the effects of increasing salinity and they observed that no growth reduction appeared at up to 100 mM NaCl and a decrease about 20% occurred at 200 mM, mostly due to the modifications in leaf morphology rather than salt toxicity. Moreover, rocket plants were able to survive at concentrations of around 300 mM.

Tolerant plants are able to use different adaptation strategies including morphological, physiological, biochemical, and molecular changes in order to cope with the salinity stress and complete their growing cycle. Salt stress is primarily perceived by the roots and the two main strategies that plants adopt for salt tolerance are the minimization of the uptake of salt by the roots and its distribution in all tissues in order to avoid the accumulation in the leaves [12]. During the osmotic-stress phase, plant growth in saline condition seems to be regulated by hormonal regulation. Among the different plant hormones, abscisic acid (ABA) is the main candidate in this mechanism since it is present in xylem sap and it increases after drought and salt stress. In the second phase, specific mechanisms controlling Na^+^ and Cl^−^ concentrations occur, such as the exclusion of salt from the phloem, its accumulation in the older leaves, in the leaf base or stems, and its compartmentalization in the vacuoles.

Shavrukov [13] pointed out the differences between salt stress and salt shock, considering salt concentration and exposure. Salt shock is considered as an extreme form of salt stress and is defined as the sudden exposition of plants to high levels of NaCl. This situation rarely occurs in natural or agricultural system where the NaCl increases gradually, but it is still applied in plant studies of salt stress responses. The osmotic and the ionic component can be also distinguished in salt shock, albeit with some differences. Indeed, when the plants are exposed to salt shock, they have to face a large difference in the osmotic pressure, causing plasmolysis and the leakage of the nutrient solution in the apoplast. Genes involved in the osmotic responses are rapidly activated in roots cells. During this phase the plants are not able to control the movement of the solutes and salt is quickly transported from the roots to the leaves.

Plant response to abiotic stresses can be described as a complex regulatory network involving different physiological pathways and interactions among signaling molecules, defense proteins, and stress-responsive genes [14]. Tolerant plants may have some peculiar stress-responsive genes which are not present in susceptible plants. Therefore, most of the research is focused on the identification of these salt tolerance associated genes and their role in the molecular mechanisms of tolerance. These genes encode various salt stress responsive proteins and transcription factors (TFs). The first ones act directly against salt stress by protecting the plants from the dehydration and regulating the biosynthesis of osmo-protectants, while the latter are involved in the regulation of the gene expression and the signal transduction.

TFs have different domains, a transcriptional domain and DNA binding domains. TFs bind to specific *cis* regulating DNA sequence modulating the expression of salt-responsive genes. Several TFs share the same binding domain and on the basis of this sequence they are classified in families, such as NAM-ATAF1.2-CUC (NAC), APETALA2/Ethylene Responsive Factor (AP2/ERF), Basic Leucine Zipper Domain (bZIP), MYB, and WRKY [15]. Cavaiuolo et al. [16] performed a transcriptomic analysis of short-term acclimation in *Diplotaxis tenuifolia* L. after salt shock exposition and identified 20879 active genes and 12995 silent genes in response to stressful conditions. Among these, 29 TFs were upregulated and 44 downregulated under salinity, with NAC, AP2/ERF, and bZIP families being the most represented.

The study of TFs role, their regulation, and the target genes represents one of the modern genetic engineering strategies to improve crop tolerance against abiotic stresses. TFs are considered to be a good target since most of them are early responsive genes under stress condition and they are able to control a set of genes involved in plant stress resistance [17,18]. This approach reveals the complexity of transcriptional regulation networks of plant genes since an overlap in the TFs expression in responses to multiple abiotic stresses often appears. Indeed, besides the regulation of stress responses, TFs participate in different biological and physiological processes, such as plant development or senescence, regulating a cluster of downstream target genes [19,20]. Thus, linking specific TFs with a single stress response in a big network of pathways is a big challenge.

Beside the study of plants stress responses mechanisms, transcriptome analysis is a strategy more frequently used to understand the effects and the mode of action of biostimulants on plants [21,22,23,24].

The object of this study was to evaluate the response of *Diplotaxis tenuifolia* L. to salt stress and the effect of an aqueous borage extract as biostimulant treatment to counteract salinity stress. The expression of some of the TFs typically involved in salt stress response was studied. Moreover, plant response was analyzed in terms of change in different physiological parameters, such as chlorophyll, chlorophyll *a* fluorescence, carotenoids, phenols, and anthocyanins. The borage extract used in this experiment showed positive effect both on primary and secondary metabolism of lettuce and rocket plants grown under non-stressful conditions in previous experiments [25].

## 2. Results

### 2.1. Gene Expression Analysis

The quality and concentration of RNA extracted from rocket leaves subjected to salt stress and treated with borage extract were measured using the NanoDrop instrument. On average, the A260/280 ratio of RNA samples was 2.30 and the ratio of A260/230 was 2.20, indicating that the RNA was not contaminated by proteins or phenols.

Selected primers were preliminary tested in a qRT-PCR in order to check if they bind and amplify the RNA via melting curve analysis. The results obtained showed that all primers worked successfully.

**Figure 1:**
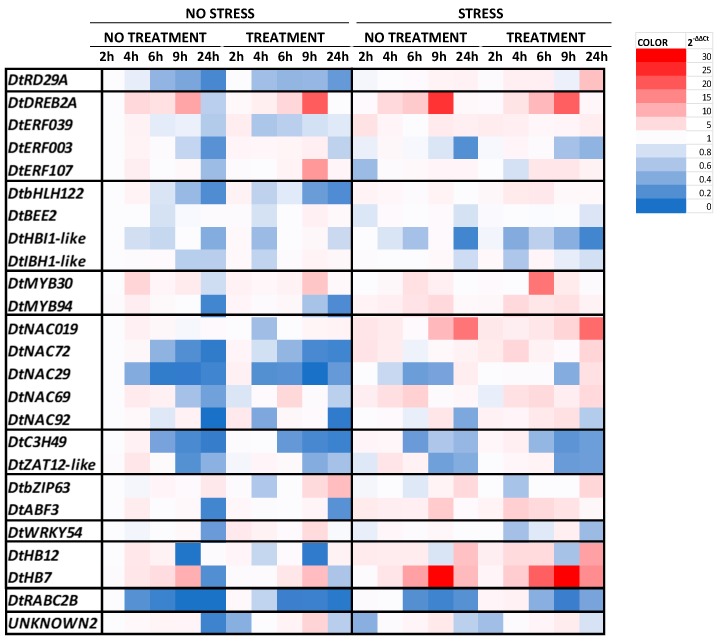
Heatmap showing temporal expression (2^−ΔΔCt^) of selected transcription factors associated with salt stress responses in rocket plants grown under salt stress condition and treated with a borage extract. The rows represent the transcription factors and within each row, the blue shaded areas indicate downregulated gene expression, whereas the red shaded areas indicate upregulated gene expression.

The changes in the expression of the transcription factors involved in the salt stress response are clustered into a heatmap (Figure 1). The results of statistical analysis and the graphs representing the expression analysis of each transcription factor are reported in the Supplementary Table S1 and Figures S1–S11. Different trends in the transcript levels were found in response to salt stress, borage treatment, and over time. Under no stress condition a similar pattern of expression was observed for *DtRD29A*, *DtbHLH122*, *DtNAC72*, *DtNAC29*, *DtC3H49*, and *DtRABC2B* with a progressive decrement within 24 h. Their trends did not change in response to borage treatment. The minimum level was observed for *DtNAC29* after 9 h and for *DtRABC2B* after 24 h. In contrast, a constant increment of the expression levels was observed for *DtDREB2A* and *DtHB7* within 9 h. After 24 h the expression of almost all of TFs was downregulated in plants grown under non-stress condition, regardless of the treatment. In contrast, stressful growing condition induced a general increase in the expression levels of TFs, as shown by the abundance of the red color in the right side of the heatmap (Figure 1). In particular, an induction of the expression levels was observed in response to salt stress after 6 h and 9 h.

A constant decrease in the expression levels of *DtRD29A* was found in non-stressed leaves within 24 h, both in treated and control plants. An opposite trend was detected in rocket grown under salt stress conditions; a gathering but slight increase was observed in control plants, whereas the expression of *DtRD29A* rose faster in plants treated with borage extract. In particular, a constant increase was observed within 6 h, followed by a decrease at 9 h and a final growth after 24 h. At this time point, the 2^−ΔΔCt^ value of treated plants was almost eight times larger than the initial point (non-stressed and non-treated at 2 h).

The gene expression of the selected transcription factors belonging to AP2/ERF family showed different patterns in response to the experimental conditions. The expression levels of *DtDREB2A* increased within 9 h and decreased at 24 h in untreated and unstressed plants. This trend did not change in response to the application of the borage treatment or after the exposition to high salinity. Only few differences appeared in specific time points. In particular, the expression was higher (+72%) in plants treated with borage extract at 9 h under no stress condition if compared with the control ones at the same time point. In contrast, the highest level was reached in control plants grown under salt stress condition, and the expression resulted almost 24-fold higher than the calibrator (non-stressed and non-treated at 2 h).

The expression levels of *DtERF107* and *DtERF039* in unstressed and untreated plants were generally low. The expression of *DtERF107* did not change in response to borage treatment, except after 9 h where the 2^−ΔΔCt^ value increased more than 12 times. Salt stress slightly induced the accumulation of *DtERF039* mRNA in all samples, regardless of the treatment.

*DtERF003* expression levels were very low in control plants both under non-stressed and stressed conditions. Two different trends emerged in plants treated with the borage extract depending on the stress condition. In particular, under optimal condition borage treatment led to three times increase in the expression levels by within 9 h, whereas salt stress generally lowered all values and the trend observed in treated plant was similar to that of control.

The expression levels of selected transcription factors belonging to the bHLH family were very low and only few differences were observed in response to stress or borage treatment. The expression of *DtbHLH122* in control plants grown under non-stressful conditions was double at 4 h compared with the control, then constantly decreased after 24 h. Borage treatment affected the trend anticipating the peak at 2 h. Salt stress generally induced the expression of this TF, mostly after 4 and 6 h in plants treated with borage extract.

No change was observed in the expression of *DtBEE2* in response to stress or treatment, only a slight increase was detected in non-stressed and treated plants after 9 h.

Different patterns were detected in the expression of *DtHBI1-like*. In particular, a constant decrease to 0.2 was observed within 24 h in response to salt stress both in control and in treated plants. No clear effect related to salt stress, time, or treatment appeared in the expression of *DtIBH1-like.*

Under non-stressful condition the expression of *DtMYB30* in control plants was similar to the calibrator and only a peak appeared at 4 h. In plants treated with borage extract the expression increased within 9 h reaching the 2^−ΔΔCt^.value of 7.4, then decreased at 24 h. Salt stress did not affect the expression of *DtMYB30,* only a peak in the expression appeared in combination with borage treatment after 6 h. The expression levels of *DtMYB94* in control plants showed a similar pattern of those observed in the expression of *DtMYB30* with an increase at 4 h followed by a constant decrease up to 0.2 at 24 h. Similar trend was found also in plants treated with borage extract. Salt stress generally induced the expression levels mostly after 4, 6, and 9 h, both in non-treated and treated samples.

Different expression patterns were found in response to the experimental condition from the analysis of the TFs belonging to the NAC family. As previously mentioned, a constant decrease was observed within 24 h in the expression levels of *DtNAC29* and *DtNAC72* of plants grown under non-stressful condition, regardless the treatment. Under salt stress they showed two different trends. In particular, within 9 h the salt stress did not affect the expression of *DtNAC29* in untreated plants while the expression slightly increased in treated plants. After 24 h the expression levels increased by four and three times, in treated and non-treated samples, respectively. The expression of *DtNAC72* was induced four times after 2 h of stress in control plants. Afterwards, the amount of transcript decreased within 6 h and slightly increased at 24 h. The expression of *DtNAC69* in untreated and unstressed plants increased more than three times at 4 h, then progressively decreased within 24 h. The accumulation of the transcripts was not affected by the borage treatment, except after 6 h, when a peak more than five times higher appeared. In contrast, salt stress generally induced their accumulation. The expression levels of *DtNAC92* in control plants grown under non-stressed condition increased more than double within 9 h and subsequently decreased at 24 h. The expression levels of *DtNAC019* were similar to those observed in *DtRD29A.* Indeed, under non-stressed condition the expression did not change during time or in response to borage treatment. In contrast, salt stress induced a constant increase in all samples. In particular, values increased from a 2^−ΔΔCt^.value of 4 to almost 18 after 24 h of exposure to salt stress.

A similar pattern of expression was observed for *DtC3H49* and *DtZAT12-like*. In particular, *DtC3H49* was strongly downregulated after 6 h in all growing conditions, whereas after 2 and 4 h of salt stress exposition, the expression slightly increased both in control and in treated plants. In a similar way, *DtZAT12-like* expression was downregulated both under stress and non-stress conditions at 9 h and 24 h.

The expression of *DtABF3* was generally low and all values ranged from 1 to 3 within 9 h in plants grown under non-stressful condition. After 24 h the expression was downregulated, regardless of the borage treatment. Salt stress induced a general increase in the expression of *DtABF3* within 9 h. In general, no change was observed in the expression of *DtbZIP63* within 6 h, neither in control nor in treated plants and regardless of the growing conditions. Under non-stressful conditions, the expression of *DtbZIP63* was three times higher at 24 h in control plants, whereas it was strongly upregulated in treated plants after 9 h and 24 h and the values were 5 and more than eight times higher than the calibrator.

The expression levels of *DtWRKY54* in control plants were generally low within 24 h, both in non-stressed and in stressed conditions. In contrast, borage treatment slightly induced its accumulation, mostly under a non-stress condition. After 2 h the 2^−ΔΔCt^ value was three times higher than control but it constantly decreased during time, apart from a peak after 9 h. Under salt stress the expression levels were reduced and the highest value of around 3 was found in treated plants after 9 h of exposure to high salinity.

The expression of both *DtHB12* and *DtHB7* increased in response to salt stress. In particular, the expression of *DtHB12* in plants grown under non-stressful condition did not change during time and the maximum level was reached in control plants after 4 h. After 9 h the TF was downregulated, both in treated and control plants. The same effect was observed in plants grown under salinity. The maximum levels were reached after 24 h of exposure both in control and in treated plants; the values were eight and 12 times higher than the internal target, respectively. The expression pattern of *DtHB7* was very similar to those of *DtHB12* in control and treated plants. Moreover, expression levels increased in response to salt stress, but the trend did not change. The maximum value was reached after 9 h of salt exposure and the expression was almost 30 times higher than the internal calibrator, both in control and in treated plants.

The expression levels of *DtRABC2B* in rocket leaves revealed a general downregulation within 24 h. In particular, under non-stressed condition the 2^−ΔΔCt^ value of control plants dropped to 0.2 after 4 h and reached the minimum point (0.04) after 24 h. A similar trend was found in plants grown under salt stress, even if the decrease started 2 h late. Plants treated with borage extract showed higher values, mostly in combination with salt stress.

The expression of an unknown transcription factors named *Unknown2* grew for the first 6 h in unstressed and untreated plants, then decreased to 0.6 at 24 h. Borage treatment slightly led to a decrease of the expression level at 2 h whereas a peak almost seven times higher emerged after 9 h. Under salt stress, both control and treated plants had the same trend excluding at 24 h.

### 2.2. Physiological Analyses

#### 2.2.1. Chlorophyll

**Figure 2:**
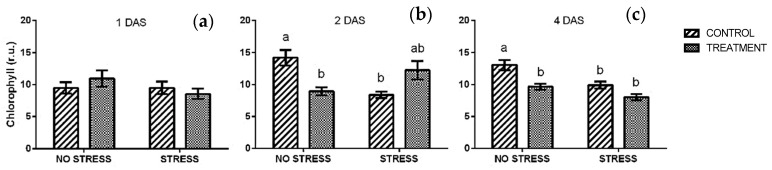
Chlorophyll content determined in vivo in rocket leaves treated with water (CONTROL) and with borage extract (TREATMENT) and subjected to salt stress (200 mM). Measures were taken after one day (**a**), two days (**b**)**,** and four days (**c**) of stress. Values are means ± SE (*n* = 11). Data were subjected to two-way ANOVA. Different letters, where present, represent significant differences (*p* < 0.05).

The chlorophyll content measured in vivo did not show any significant change one day after the beginning of the stress (Figure 2a), not in response to salt, nor to borage treatment. All values were around 10 r.u. A significant interaction between stress and treatment was observed after two days of stress (Table S2). In particular, under a non-stress condition, the chlorophyll content of plants treated with borage extract was significantly lower than control while an opposite effect was found under a stress condition (Figure 2b). After four days (Figure 2c) chlorophyll content was similar in stressed plants (regardless of the treatment) and non-stressed plants treated with borage extract. However, their values were significantly lower than those observed in control plants grown without salt stress.

Chlorophyll content showed different trends during the experimental time course: under a non-stress condition, chlorophyll content increased in control plants from 10 r.u. to almost 15 r.u., while it did not change in plants treated with a borage extract. Stressed plants did not show any variation over time, only a slight increase was observed after two days in plant treated with borage extract (Figure 2b).

**Figure 3:**
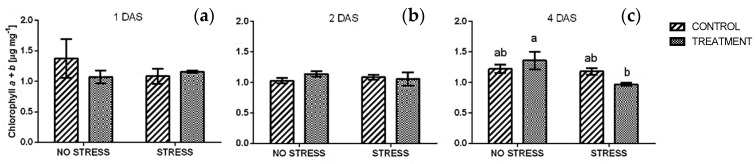
Chlorophyll *a + b* concentration in rocket leaves treated with water (CONTROL) and with borage extract (TREATMENT) and subjected to salt stress (200 mM). Measures were taken after one day (**a**), two days (**b**), and four days (**c**) of stress. Values are means ± SE (*n* = 4). Data were subjected to two-way ANOVA. Different letters, where present, represent significant differences (*p* < 0.05).

Chlorophyll concentration obtained with the destructive method (Figure 3) did not show the same trend of the in vivo analyses. In particular, no significant change appeared in chlorophyll *a + b* concentration of rocket leaves after one and two days of stress (Figure 3a and b) and the average values were around 1 µg mg^−1^ FW. In contrast, salt stress induced a significant decrease in the concentration of chlorophyll *a + b* in plants treated with borage extract after four days of stress (Figure 3c).

#### 2.2.2. Total Carotenoids

**Figure 4:**
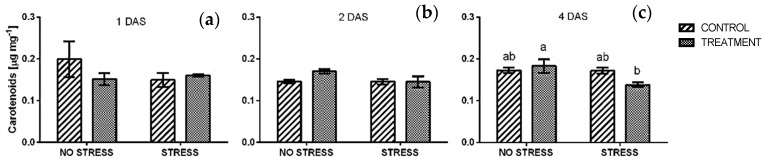
Carotenoids concentration in rocket leaves treated with water (CONTROL) and with borage extract (TREATMENT) and subjected to salt stress (200 mM). Measures were taken after one day (**a**), two days (**b**)**,** and four days (**c**) of stress. Values are means ± SE (*n* = 4). Data were subjected to two-way ANOVA. Different letters, where present, represent significant differences (*p* < 0.05).

The concentration of carotenoids (Figure 4) in rocket leaves showed the same trend observed in chlorophyll *a + b* analyses. In particular, no significant change was detected after 1 and two days of stress and all values were similar to non-stressed control (Figure 4a,b). After four days of stress, carotenoids level of plants treated with borage extract significantly decreased, while it did not change in control plants (Figure 4c).

#### 2.2.3. Phenolic Index and Anthocyanin

Phenolic index and anthocyanin content measured in rocket leaves are listed in Table 1. Both parameters were not affected by the salt stress, in any of the time points analyzed. Moreover, statistical analysis did not detect any significant difference between samples in response to borage treatment (Table S2).

#### 2.2.4. Chlorophyll a Fluorescence

**Figure 5:**
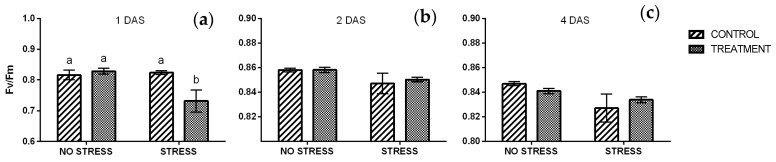
Maximum quantum efficiency of PSII (Fv/Fm) measured in rocket leaves treated with water (CONTROL) and with borage extract (TREATMENT) and subjected to salt stress (200 mM). Measures were taken one day (**a**), two days (**b**)**,** and four days (**c**) of stress. Values are means ± SE (*n* = 9). Data were subjected to two-way ANOVA. Different letters, where present, represent significant differences (*p* < 0.05).

The maximum quantum efficiency of PSII (Fv/Fm) gives an information on the plant’s potential photosynthetic ability and can be used as stress marker since its sensitivity to different stressful conditions. Generally, an average value of 0.83 is considered the stress threshold for herbaceous plants, whereas lower values indicate stressful conditions for the plants with limitation of physiological processes [26]. A significant interaction between stress condition and treatment was observed after one day of stress (Table S2). Fv/Fm values were really close to the threshold in non-stressed plants and the same value was observed in control plants grown under salt stress. In contrast, stressed plants treated with borage extract had a lower value of about 0.73 (Figure 5a). After two and four days of stress (Figure 5b,c) the Fv/Fm ratio increased exceeded the 0.83 threshold, regardless of the stress condition or the treatment.

**Figure 6:**
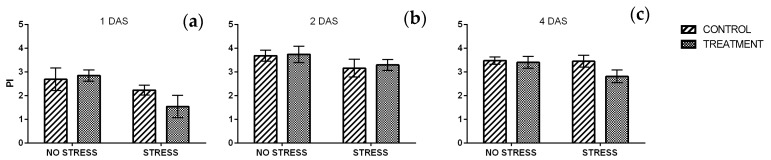
Performance index (PI) measured in rocket leaves treated with water (CONTROL) and with borage extract (TREATMENT) and subjected to salt stress (200 mM). Measures were taken after one day (**a**), two days (**b**)**,** and four days (**c**) of stress. Values are means ± SE (*n* = 9). Data were subjected to two-way ANOVA. Different letters, where present, represent significant differences (*p* < 0.05).

The performance index (PI) did not show any significant change, not in response to salt stress, neither to treatment. After one day of stress (Figure 6a) the results had a similar trend to Fv/Fm ratio and stressed plants treated with borage extract had a lower value if compared with the other conditions. With the exception of this, all of the values averaged from 2.2 to 2.8. After two and four days of stress the PI increased in all samples and values averaged from 2.8 and 3.7 (Figure 6b,c). Salt stress significatively affected the PI, however no significant difference emerged among samples (Table S2).

#### 2.2.5. Nitrate

**Figure 7:**
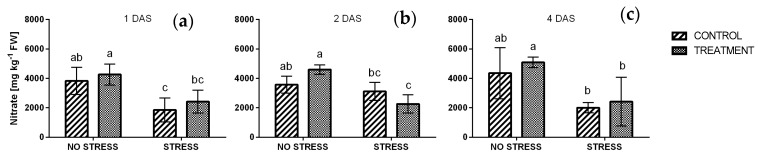
Nitrate concentration in rocket leaves treated with water (CONTROL) and with borage extract (TREATMENT) and subjected to salt stress (200 mM). Measures were taken after one day (**a**), two days (**b**) and four days (**c**) of stress. Values are means ± SE (*n* = 4). Data were subjected to two-way ANOVA. Different letters, where present, represent significant differences (*p* < 0.05).

In general, salinity significantly caused a decrease of the nitrate concentration in rocket leaves, regardless the treatment. After one day (Figure 7a) nitrate contents of non-stressed plants were 3836 and 4264 mg kg^−1^ FW in control and after borage treatment, respectively. At the same time nitrate levels were halved under stress condition. In particular, nitrate concentrations were 1856 mg kg^−1^ FW in control plants and 2423 mg kg^−1^ FW in plants treated with borage extract. A similar effect has been observed also after four days, while after two days, a significant interaction between stress and treatment was detected (Table S2).

#### 2.2.6. Reducing and Total Sugars

**Figure 8:**
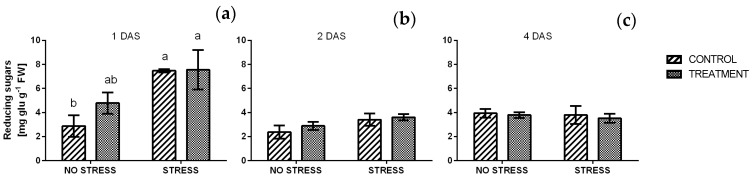
Reducing sugars concentration in rocket leaves treated with water (CONTROL) and with borage extract (TREATMENT) and subjected to salt stress for 24 h. Measures were taken after one day (**a**), two days (**b**) and four days (**c**) of stress. Values are means ± SE (*n* = 4). Data were subjected to two-way ANOVA. Different letters, where present, represent significant differences (*p* < 0.05).

**Figure 9:**
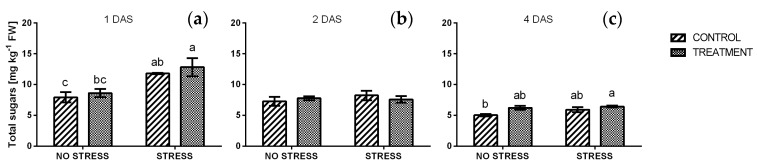
Total sugars concentration in rocket leaves treated with water (CONTROL) and with borage extract (TREATMENT) and subjected to salt stress (200 mM). Measures were taken after one day (**a**), two days (**b**)**,** and four days (**c**) of stress. Values are means ± SE (*n* = 4). Data were subjected to two-way ANOVA. Different letters, where present, represent significant differences (*p* < 0.05).

A significant effect of salinity was detected one day after the beginning of the stress (Figure 8a) when the average value of reducing sugars increased from 2.9 mg g^−1^ FW in non-stress condition to 7.5 mg g^−1^ FW under salt stress. A slight but not significant increase was also observed in non-stressed conditions, in response to borage treatment. After two and four days of stress the concentration of reducing sugars decreased and no significant differences were observed among samples. In particular, all values were similar to control plants grown in non-stressful condition and values ranged from 2.4 to 3.9 mg g^−1^ FW. A similar trend was found in the concentration of total sugars, as reported in Figure 9.

#### 2.2.7. Lipid Peroxidation

**Figure 10:**
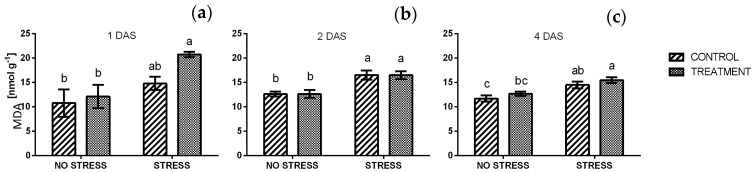
Concentration of malondialdehyde (MDA) in rocket leaves treated with water (CONTROL) and with borage extract (TREATMENT) and subjected to salt stress (200 mM). Measures were taken one day (**a**), two days, (**b**) and four days (**c**) of stress. Values are means ± SE (*n* = 4). Data were subjected to two-way ANOVA. Different letters, where present, represent significant differences (*p* < 0.05).

The level of lipid peroxidation in control plants grown under non-stressed conditions was around 12 nmol g^−1^ FW at each time point, and a similar value was observed in response to borage treatment. After one day of stress (Figure 10a) a significant difference was found between plants treated with borage extract under stressed and non-stressed condition, while only a slight increase was observed in control plants. In particular, the highest level of lipid peroxidation was found in plants treated with borage extract and grown under salt stress (20.7 nmol g^−1^ FW). After two and four days, the level of lipid peroxidation in stressed samples slightly decreased to about 15 nmol g^−1^ FW. However, it remained significantly higher than non-stressed samples.

#### 2.2.8. Osmolytes

**Figure 11:**
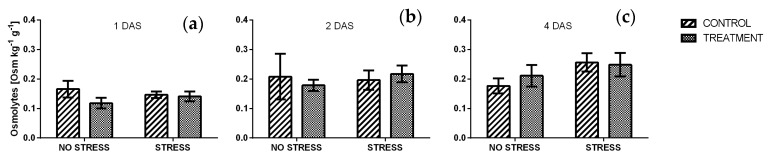
Osmolytes concentration in rocket leaves treated with water (CONTROL) and with borage extract (TREATMENT) and subjected to salt stress (200 mM). Measures were taken after one day (**a**), two days (**b**)**,** and four days (**c**) of stress. Values are means ± SE (*n* = 4). Data were subjected to two-way ANOVA. Different letters, where present, represent significant differences (*p* < 0.05).

The levels of osmolytes in rocket leaves (Figure 11) was not affected by the stress condition or by the borage treatment, in any of the time point analyzed. In particular, after one day of salt stress, all values averaged from 0.12 and 0.17 Osm kg^−1^g^−1^. A slight increase appeared after two and four days (Figure 11b,c), however no significant difference was detected among the samples.

#### 2.2.9. Abscisic Acid

**Figure 12:**
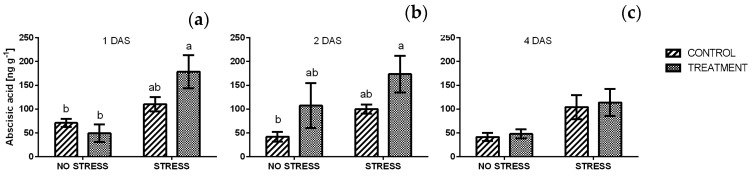
Abscisic acid concentration in rocket leaves treated with water (CONTROL) and with borage extract (TREATMENT) and subjected to salt stress (200 mM). Measures were taken one day (**a**), two days (**b**) and four days (**c**) of the stress. Values are means ± SE (*n* = 4). Data were subjected to two-way ANOVA. Different letters, where present, represent significant differences (*p* < 0.05).

After one and four days (Table S2) a significant effect of the stress was found in the ANOVA. Initially, the average concentration of ABA in non-stressed samples was around 60 mg g^−1^ and no significant effect was detected after the application of the borage extract. The ABA contents in plants grown under stress conditions were significantly higher than in non-stressed ones, and averaged from 110.4 to 178.5 mg g^−1^. The highest level was reached in samples treated with borage extract and it was three times higher than in non-stressed samples (Figure 12a). After two days of stress (Figure 12b) the concentration of ABA did not change in almost all samples if compared with the previous time point. Moreover, the ANOVA showed a significant effect of treatment (Table S2). After four days the concentration of ABA in all samples decreased, and no significant difference was detected even if ABA levels in stressed samples were higher than non-stressed ones (Figure 12c).

## 3. Discussion

*Diplotaxis tenuifolia* can be considered as salt tolerant species due to its ability to maintain considerable growth rates under increasing salinity without any relevant variation to physiological parameters [11,27]. In the present study, rocket plants have been exposed to 200 mM NaCl for four days, just before the harvest, in order to evaluate the plant responses to salt shock with no acclimatization period. To better understand the nature of the different processes activated to cope the stress, a transcriptome analysis and the evaluation of different physiological and biochemical mechanisms in biostimulant-treated and not-treated rocket plants were analyzed.

At a molecular level, the transcriptional regulation depends on the interaction between transcription factors and a broad range of target genes involved in different stress responses pathways. TFs are involved in the primary stress responses, through the repression/activation of a set of genes associated with abiotic stress responses during the first few hours of stress exposure. For these reasons, 24 transcription factors belonging to different families and a stress-related gene (*RD29A*) were chosen and their expression within 24 h of salt stress was analyzed. Since TFs expression is dynamic and often transient, the measure at one single time point is not enough to understand their activity, thus their expression over time after 2, 4, 6, 9, and 24 h was evaluated. Most of the genes studied in this work showed a trend in their expression that might suggest a circadian control.

*RD29A* is considered as a stress-response marker gene and has been studied in response to different abiotic stress treatments. In this experiment the *DtRD29A* expression constantly decreased under control conditions, both in untreated and treated plants. These values are consistent with those reported in another study, in which the gene was not expressed at significant levels during normal plant growing conditions [28]. As expected, *DtRD29A* transcripts increased under salt stress conditions, whereas its expression was particularly affected by the combination of NaCl and borage treatment. This result might suggest that rocket plants treated with borage extract were more sensitive to salt stress than the non-treated ones. Interestingly, Lee et al. [29] observed that *RD29A* expression pattern changed when exposed to single or combined (salt and ABA) treatments and the combination of the inputs led to unique dynamic behaviour that cannot be explained by the sum of the single responses. Li et al. [30] proposed an interaction between an ABRE binding protein (ABF3) and another transcription factor (NAC72) in the regulation of the expression of RD29A.

In our experiment, a rapid increase of *DtNAC72* expression induced by salt exposure, similar to that reported by Tran et al. [31] in *Arabidopsis thaliana* exposed to 250 mM NaCl for 24 h was observed. Fujita et al. [32] observed a slower increase of the expression over time, probably due to the lower concentration of NaCl used in their experiment. Moreover, the same authors reported the existence of two different pathways for the *NAC72* expression, one ABA-dependent in non-stressful condition, and the other ABA-independent under salt stress. It might explain why borage treatment induced the expression of *DtNAC72* under salinity and not under normal growing conditions. In contrast, a gradual increase appeared in the expression of *DtNAC019* in response to salt stress but in this case borage extract did not show any effect. Even if *NAC019* and *NAC72* are homologs, their response to salt and biostimulant treatment is quite different. Results obtained in the present study confirmed that both TFs are involved in stress response and may act in two different pathways.

Similar to *DtNAC72*, the expression of *DtABF3* was rapidly affected by salt stress. ABF3 plays an important role in ABA signaling both in normal and stressful conditions and it is induced by ABA and osmotic stress, as can be seen in reference [33].

MYB is a large family of transcription factors that are well-known to be involved in drought responses [34]. In this experiment the expression of two MYB TFs, *DtMYB94* and *DtMYB30* was examined. The expression of *DtMYB94* constantly increased in response to salinity and was similar to those results reported for Arabidopsis [35]. At the same time, *DtMYB30* expression showed a different pattern in response to salt and borage treatment. In general, its expression was lower in plants grown under stress. Recent studies reported that both *MYB30* and *MYB94* are involved in the activation of cuticular wax biosynthesis [35,36]. Moreover, *MYB30* acts as a positive regulator of ABA signaling response [37], in the accumulation pattern of very-long-chain fatty acids such as waxes, phospholipids, and complex sphingolipids [38], and promoting the expression of a subset of brassinosteroids (BRs) target genes [39,40]. As mentioned above, an increase in lipid peroxidation was found in plant grown under salt stress and treated with borage extract. These finding may suggest that rocket plants reacted to salt stress through the accumulation of cuticular waxes and the production of new lipids molecules.

*WRKY54* as well as *MYB30* are involved in the BRs signaling pathway [41]. *DtWRKY54* was expressed at low levels in control and untreated plants and was slightly induced by salt stress. A similar result was observed in *Arabidopsis* by Zhou et al. [42]. Moreover, the authors reported an increased tolerance to salt in transgenic soybean plants over-expressing *WRKY54* gene. In rocket plants treated with borage, extracting the expression of Dt*WRKY54* was induced under normal growing conditions, whereas it caused a general downregulation in plants exposed to salinity. Since *WRKY54* co-operate with *WRKY70* as negative regulators of the plant response to osmotic stress [43], so the lower expression levels observed in plants treated with borage and then exposed to salt stress might suggest a better tolerance to osmotic stress.

Rocket plants grown under salt stress showed high levels in the expression of *DtHB7* and *DtHB12*. Both TFs belong to the HD ZIP family and are negative regulators of ABA signaling by acting as positive regulators of a protein phosphatase (PP2C) genes [44]. *DtHB7* was more upregulated than *DtHB12*, confirming what was observed by Zimmermann et al. [45]. Moreover, plants overexpressing *AtHB7* showed a higher chlorophyll content [46], and this might be linked to the high chlorophyll levels observed in rocket plants grown under salt stress.

*ERF003*, *ERF107,* and *ERF39* are members of the ERF transcription factor subfamily and they are involved in abiotic stress responses through binding the ethylene-responsive element (ERE) [47]. Their expression has been reported to increase in response to high concentration of salt and to contribute to salt and drought tolerance [48,49,50]. In this study *DtERF107* and *DtERF39* expressions were induced in plants exposed to salt stress, in accordance with what is reported above. In contrast, the expression of *DtERF003* rapidly increased in response to borage treatment but no changes appeared in plants exposed to salinity, suggesting that in rocket *DtERF003* is not involved in salt stress response.

An overall upregulation of *DtbHLH122* expression was found in rocket plants exposed to salt stress. *BHLH122* has been reported to be a positive regulator of drought and osmotic stress signaling resistance and its expression is at least partly independent from ABA signaling [51]. The same authors reported that *Arabidopsis* plants overexpressing *bHLH122* showed an increased resistance to water, salt and osmotic stresses. Moreover, bHLH122 was able to repress a gene involved in ABA catabolism, leading to the accumulation of ABA. These results might explain the increased content of ABA observed in rocket plants grown under salt stress and treated with borage extract.

Besides *bHLH122*, the expression of other three TFs of bHLH family: *BEE2*, *HBI1,* and *IBH1,* all of which are involved in the brassinosteroids signaling pathway, was also analyzed. *BEE2* and *HBI1-like* are induced by BRs and are repressed by ABA, while *IBH1* is reported to inhibit both *BEE2* and *HBI1*. Moreover, plants overexpressing *IBH1* showed a decreased stress response [52]. All TFs were generally expressed at very low levels in rocket plants regardless the growing condition. These results, together with the low expressions levels of *DtMYB30* and *DtWRKY54* and the up regulation of *DtNAC72* (a negative regulator in the BRs signaling pathway) might suggest that for the specific condition tested in this study, stress responses in rocket plants are mediated more through ABA signaling pathways than through brassinosteroids signaling.

This is further confirmed by the increased concentration of ABA observed in rocket plants grown under salt stress. It is also known that an antagonistic interaction between ABA and BRs exists [53].

*bZIP63* is a transcription factor involved in the processes regulating the circadian phase through the regulation of low-energy response. It is a target of a sugar-sensing kinase (*SnRK1*), a conserved gene usually activated during starvation [54,55]. In our experiment, we observed that *DtbZIP63* expression was low in control plants and only a peak was observed after 24 h. This time point corresponds to 8:00 am, and the accumulation of *DtbZIP63* transcripts might be linked to a low concentration of sugars at night. Indeed, it has been reported that *bZIP63* expression is usually repressed by sugars and ABA [56]. Interestingly, borage extract induced the expression of the gene under non-stressful conditions, even if the level of sugars and ABA in rocket leaves did not change. Moreover, *bZIP63* is reported to function as a negative regulator of osmotic stress tolerance in *Arabidopsis* during seeds germination [57], thus the low expression detected in stressed plants might also confirm the mechanism of tolerance of rocket plants.

RABC2B is a protein involved in signal transduction and intracellular transport. In rocket plants *DtRABC2B* was generally expressed at low levels in both growing conditions, in contrast to those reported by Liu et al. [58], who observed an induction in response to salt stress in *Arabidopsis*. Borage treatment induced *DtRABC2B* expression, mostly after 2 and 4 h of stress exposition. However, to date there has been scarce information about the biological role and the regulation of these proteins in plants.

*NAC92*, *NAC29,* and *NAC69* are involved in multiple abiotic stress responses. Their expression levels were generally low in leaves under non-stress conditions, as reported also by Xue et al. [59], and were induced by salt stress at different time points.

The trend of expression of the unknown transcription factor was similar to those observed in *DtHB7* and *DtDREB2A*. Moreover, as well as *DtBEE2*, *DtWRKY54,* and *DtERF107*, a peak of transcript appeared after 9 h.

An overall upregulation of the gene expression appeared in response to salt stress conditions. In general, the results obtained in the present experiment confirmed those reported in the transcriptome for most of the TFs analyzed. Novel findings emerged from the *bZIP63*, *ERF107*, *DREB2A,* and *NAC92* expression. Moreover, the analysis of the gene expression over time allowed us to see the different trends in the TFs. For example, the expression levels of C3H49 and RABC2B were higher in plants exposed to salt stress than with the unstressed controls after 24 h, as reported by Cavaiuolo et al. [16]. However, the trend of their expression shows a constant decrease during time.

The content of chlorophyll and the chlorophyll fluorescence-related parameters did not significantly change in response to salt stress. These results are consistent with those reported in other studies, in which salt stress did not cause significant alteration in photosynthetic apparatus after few days of exposition to stressful conditions [60,61,62]. It is known that these parameters are considered as biochemical markers of salt tolerance, since chlorophyll levels and PSII efficiency usually decrease quickly in sensitive plants [63]. The content of chlorophyll in green leafy vegetable is also important because it defines the visual appearance of the product and influences the consumer choice [64]. The application of biostimulant products has been shown to increase the biosynthesis of chlorophyll in different vegetables [65,66].

This experiment confirms that *D. tenuifolia* is a salt tolerant crop [11] due to its ability to counteract a sudden exposition to a high level of salinity, at least under our experimental conditions. At the same time, the short treatment time with the borage extract did not increase the chlorophyll content as well as the PSII efficiency in rocket plants grown under salt and non-salt conditions. In contrast, after one day of exposition to salt stress plants treated with borage extract showed a significant decrease of Fv/Fm ratio. Since this value did not change in untreated plants in response to salt treatment and in plant treated with the borage extract grown in non-stressful condition, it may indicate that treatment induced the plant temporarily susceptible to salinity. The same effect observed in chlorophyll concentration was found also in carotenoids level and it makes sense because of their role as accessory light-harvesting pigments and protecting chlorophyll molecules. These results were compatible with those obtained by Bulgari et al. [25] after two applications of the same borage extract on rocket and lettuce plants grown under non-stressful conditions. No significant modification of chlorophyll and carotenoids content were also detected in *Eruca sativa* Mill. grown under salt stress, as reported by Barbieri et al. [67].

A moderate salinity has a positive effect increasing the concentration of phytochemicals, such as flavonoids, phenolic acids, and tannins [68,69,70,71]. These polyphenolic compounds play an important role in plant protection against reactive oxygen species (ROS), which are produced in plant tissues when physiological metabolism is impaired by various environmental stresses.

Besides their roles in plants, phenolic compounds exert an important function for human health due to their potential health benefits [72].

Several biostimulant products were reported to enhance the secondary metabolism and increase the synthesis of phytochemicals [73]. Borage extracts have shown to be effective in improving total phenols, flavonoids, and antioxidant capacity in lettuce plants [25]. This was not the case in the present study, as no significant changes were recorded, not in response to salt, not to treatment. A high variability in total phenols concentration was also observed by Hamilton et al. [74] within species and between trials. The different response obtained in our experiment compared to those reported by Bulgari et al. [25] by applying the same extract might be due to the number of applications or to genetic diversity of the treated plants. Moreover, the imposition of a significant level of salt might have “hidden” the effects of the borage extract observed in other experiments, probably related to the presence of molecules involved in the activation of signaling or exerting a hormone activity.

A significant increase of total sugars appeared in leaf tissues after one day of salt exposition, both in treated and non-treated plants. Afterwards, values declined to non-stress levels and remained stable until harvest (four days of salt stress). This trend may be related to the increased reduction of sugar concentrations observed. The accumulation of sugars has been reported in different plants species exposed to salinity [75,76]. Carbohydrates are products of photosynthesis and they are the building blocks and source of energy for plant growth. Besides these roles, sugars are involved in several processes related to plant stress responses, acting as signaling molecules, osmo-protectants, or antioxidants [77,78]. As reported by Shavrukov [13], when plants are suddenly exposed to a high level of salinity, they suffer osmotic shock and plasmolysis, regardless of their level of tolerance. Indeed, the only difference between a sensitive and a tolerant plant is the degree of damage and the time needed to restore physiological functions. In accordance with this, a significant increase in lipid peroxidation was detected in all stressed samples. The MDA content in unstressed plants treated and non-treated, was similar to the one observed by Ozdener et al. [79] in *Eruca sativa* Mill. suggesting that the application of borage extract did not cause any damage to cell membranes.

The accumulation of osmolytes is a typical response of plant exposed to salt stress condition in order to maintain the cell turgor pressure and stabilize proteins and other cell components against denaturing effects. Plants with an improved osmolyte biosynthesis generally showed an enhanced stress tolerance [80,81,82]. In this experiment no significant change in osmolytes accumulation was observed, and this result was unexpected.

Abscisic acid is a plant hormone with several roles in plant growth and development as well as in response to abiotic stresses. It has been suggested that some responses to osmotic stress could be modulated by ABA [83]. Frike et al. [84] reported that ABA accumulates in leaf tissues more than six times within 10 min in response to an environmental stress and within the first hour following the stress event, ABA has a growth-promoting function. A slight but non-significant increase in ABA levels was observed in plants grown under salt stress. This is in line to those results observed by He and Cramer [85]. They compared the ABA accumulation in two *Brassica* species and found that ABA concentration increased more in salt-sensitive species than in tolerant ones. Results obtained in the present experiment showed a slight increase of ABA content in treated plants, mostly in combination with the salinity. This may suggest that borage extract could have a slight positive effect on the production of ABA.

Rocket is a nitrate-accumulating vegetable and it is known that nitrate content in rocket leaves changes depending on the cultivation systems as well as on the environmental conditions, such as the light intensity, photoperiod, temperature, and abiotic stresses [86,87]. In humans, after ingestion, nitrates undergo different reactions that may lead to the formation of cancerogenic compounds like nitrosamines [88]. For this reason, the European Commission Regulation established limitations on the commercialization of several leafy vegetables (EU No 1258/2011). The maximum levels of nitrate for rocket salad are 7000 mg NO_3_^−^ kg^−1^ if harvested from October to March, and 6000 mg NO_3_^−^ kg^−1^ if harvested from April to September. In this experiment, reduced nitrate content was observed in plants exposed to salinity for one, two, and four days. These findings are consistent with those reported by Barbieri et al. [67] and Urrestarazu et al. [89] studies, in which a reduction of nitrate levels in rocket and lettuce salad was found after a high salinity treatment. Strategies to decrease the concentration of nitrate are important for the production of fresh vegetables and different biostimulant products showed a positive effect reducing the nitrate levels in several crop species [66,90,91]. However, nitrate contents were not affected by borage treatment, not under control or stress condition. This result was unexpected since, in contrast, Bulgari [92] observed an increase of NR activity in vivo and a substantial reduction of nitrate concentration in rocket plants treated with the same borage extract. This variability might be due to differences in the experimental plans (period of cultivation, number of treatments, timing of the application).

## 4. Materials and Methods

### 4.1. Plant Material, Stress Treatment and Experimental Plan

The trial was carried out at the Faculty of Agricultural and Food Science of Milan in 2018. Rocket plants (*Diplotaxis tenuifolia*, L.; ISI Sementi S.P.A., Italy) were grown hydroponically into plastic tanks (35 x 25 x 20 cm) with 10 L of a modified Hoagland medium and the concentration of nutrient in the solution used was composed by 11.05 mM N, 1.4 mM P, 7 mM K, 2.19 mM Ca, 0.8 mM Mg, and 1.8 mM S, and Hoagland’s concentration for micronutrients. Seeds of rocket were manually sown into polystyrene trays filled with an agri-perlite substrate on 20 February 2018. Cultivation took place in an experimental greenhouse under controlled conditions.

The experimental design was a combination of two factors: stress and treatment, each of them with two levels. Salt stress was imposed by transferring plants to a fresh nutrient solution containing 200 mM NaCl, 35 days after sowing at 08.00 h (on March the 26^th^ 2018). The nutrient solution of non-stressed plants was also changed with a fresh one.

Treatments consisted of 20 mL of water (control) and 20 mL of a borage extract. The plant extract used in this experiment had been previously prepared and tested by our research group, as described by Bulgari et al. [25]. The borage flower extract diluted 10 mL L^−1^ has been chosen as a treatment since the positive effects obtained in this study and in previous unpublished experiments. Treatments were applied on 25 March 2018, as foliar spray onto leaves until run-off 24 h before the beginning of the stress. For gene expression analyses, leaf tissues were collected after 2, 4, 6, 9, and 24 h of exposure to salt (10.00 h, 12.00 h, 14.00 h, 17.00 h, and 8.00 h). Samples were shock frozen in liquid nitrogen before storage at −80 °C and were then used for RNA isolation. For physiological analyses, leaf tissues were collected from four biological replicates after one, two, and four days of stress. Sampled material was stored at −20 °C prior to analyses.

### 4.2. Total RNA Isolation and Analysis of Gene Expression

Starting from the *Diplotaxis tenuifolia* L. RNAseq database (SRP study accession number SRP102718) created at the University of Milan [16], the sequences of 25 genes were identified in order to be used as molecular markers for salt stress (Table S3). The sequences were selected among those showing significant changes in their expression (RPKM) in response to 24 h of exposure to salt stress conditions. Annotation for all sequences was verified by using NCBI BLAST database and their involvement in salt stress responses mechanisms has been confirmed through the literature review. Among them, 23 TFs have been identified and 1 did not show any correspondence to known gene sequences, thus it has been reported as unknown 2. Specific primers (Table S4) for all selected sequences (24 transcription factors and *RD29A*) were designed using the program Primer-Blast available at the National Center for Biotechnology Information website (https://www.ncbi.nlm.nih.gov/tools/primer-blast/).

Frozen leaves of rocket plants were thoroughly ground with liquid N using cold mortar and pestle. Approximately 100 mg were transferred to a cryotube and stored at −80 °C. Total RNA was isolated using the Spectrum Plant Total RNA Kit with on-column DNase-treatment (Sigma-Aldrich, Italy) following the steps of protocol A with a slight modification.

The concentration and the purity of RNA were assessed by measuring the absorbance at 230 nm, 260 nm, and 280 nm using a NanoDrop N-1000 spectrophotometer (NanoDrop technologies). A ratio of absorbance at 260 and 280 ≈ 2.0 is generally accepted as pure for RNA and expected 260/230 values are commonly in the range of 2.0–2.2, usually higher than the respective 260/280 value.

Three μg of RNA were reversely transcribed to cDNA using the SuperScript III cDNA Synthesis Kit according to the manufacturer’s instructions (Invitrogen, Italy).

The SYBR^®^ Green PCR Master Mix (Applied Biosystems) was used for the quantitative RT-PCR analysis. The reaction mix was prepared by adding 10 μL of SYBR Green, 0.4 μL of forward and reverse primers, 2 μL of cDNA diluted 1:20, and 7.2 μL of RNase free water. The total volume for each PCR reaction was 20 μL. Analysis was performed using the ABI7300 (Applied Biosystem) thermocycler and PCR program and reactions were run in triplicate from two biological replicates.

The expression levels were analyzed with the AB software program and results were calculated using the 2^-∆∆ct^ method described by Livak and Schmittgen [93]. According to this method, the data are presented as fold change in gene expression normalized to a housekeeping gene and relative to a calibrator. The Elongation factor 1 alpha (EF1α) was used as reference gene (housekeeping) since the ct values showed high stability in the same species grown under similar experimental conditions [61], whereas the non-stressed and non-treated sample after 2 h was chosen as an internal calibrator.

### 4.3. Physiological Analyses

#### 4.3.1. Chlorophyll

Leaves chlorophyll content was estimated in vivo using a chlorophyll content meter (CL-01 Chlorophyll Content Meter, Hansatech Instruments). The results were expressed as a chlorophyll index (relative units).

#### 4.3.2. Chlorophyll a Fluorescence

Chlorophyll *a* fluorescence was measured in vivo using a hand-portable fluorometer (Handy-PEA, Hansatech Instruments). Before all measurement leaves were dark-adapted with the leaf clips for 30–40 min. then were exposed to a saturating light (3000 μmol m^−2^ s^−1^) provided by an array of three high-intensity light-emitting diodes for 1 s. Information about the structural and functional status of photosynthetic apparatus was provided by the parameters measured, such as the maximum quantum of photosystem II (Fv/Fm) or the performance index (PI) derived from the JIP test calculation [94].

#### 4.3.3. Total Chlorophylls and Carotenoids

Chlorophylls and carotenoids were extracted from rocket leaves using 5 mL of 99.9% (v/v) methanol. Leaf disc samples (30 mg), obtained with a 5 mm diameter cork borer were kept in a dark room for 24 h at 4 °C. After that absorbance reading were measured at 665.2 and 652.4 nm for chlorophylls and 470 nm for total carotenoids with a spectrophotometer. Pigments levels were calculated by Lichtenthaler’s formula and expressed on a fresh weight basis [95].

#### 4.3.4. Phenolic Index and Total Anthocyanin

Phenolic index and total anthocyanin were determined from leaf disc samples (30 mg), obtained with a 5 mm diameter cork borer. Leaf samples were transferred to a tube containing 3 mL of methanol acidified with hydrochloric acid (1%) and were kept in dark room for 24 h at 4 °C. Absorbance readings were measured at 320 nm for total phenols, and at 535 nm for anthocyanin with a spectrophotometer. Phenolic index was expressed as Abs320 nm g^−1^ FW [96]. Anthocyanins concentration was expressed in cyaniding-3-glucoside equivalents using a molar extinction coefficient (ε) of 29,600 L M^−1^ cm^−1^ [97].

#### 4.3.5. Nitrate

Nitrate concentration was determined by the method of Cataldo et al. [98]. Fresh leaf tissue was homogenized in distilled water (1 g fresh tissue per 4 mL distilled water). The homogenate was centrifuged at 4000 rpm for 15 min at RT (ALC centrifuge-model PK130R) and the recovered supernatant was used for the colorimetric analysis. Twenty microliters of the extract were added to 80 mL of 5% (w/v) salicylic acid in concentrated H_2_SO_4_ (SA-H_2_SO_4_). Afterwards, 3 mL of 1.5 N NaOH were added. The samples were cooled to RT and absorbance at 410 nm was measured with a spectrophotometer. Nitrate content was calculated referring to a KNO_3_ standard calibration curve. Nitrate concentration was expressed as mg of NO_3_-N per kg of fresh weight.

#### 4.3.6. Reducing and Total Sugars

Reducing sugars were measured using the dinitrosalicylic (DNS) acid method. This colorimetric technique consists of a redox reaction between the 3,5-dinitrosalicyclic acid and the reducing sugars present in the sample [99]. Approximately 1 g of leaf tissue was homogenized in a mortar with 3 mL of water. The mixture was centrifuged at 4000 rpm for 15 min at RT. DNS assay was performed by mixing 0.2 mL of supernatant with 0.2 mL of DNS and incubated in a water bath at 100 °C for 5 min, then 1.5 mL of water was added to samples. After cooling at room temperature, the optical density was determined spectrophotometrically at 530 nm, using a glucose standard curve.

The total sugars were determined on the same extract using the anthrone method with slight modifications [100]. The anthrone reagent (10.3 mM) was prepared dissolving anthrone in 95% H_2_SO_4_. The reagent was left to stand for 30–40 min before use, 0.5 mL extract was placed on top of 2.5 mL of anthrone reagent incubated in ice for 5 min and then vortexed vigorously. The tubes were heated to 95 °C for 10 min and left to cool in ice. Readings were performed at 620 nm. Calibration curve was carried out using a glucose standard solution.

#### 4.3.7. Lipid Peroxidation

Lipid peroxidation was determined by measuring thiobarbituric acid reactive substances (TBARS) in accordance with the method described by Heath and Parker [101]. Approximately 1 g of leaf tissue was homogenized in a mortar with 3 mL of 0.1% (w/v) trichloroacetic acid (TCA). The mixture was centrifuged at 4500 rpm for 10 min at room temperature. TBARS assay was performed by mixing 1 mL of supernatant with 4 mL of 20% (w/v) TCA, 25 µL of 0.5% thiobarbituric acid (TBA) and incubated in a water bath at 95 °C for 30 min. After being cooled on ice, the tubes were centrifugated at 4000 rpm for 10 min and the optical density was determined spectrophotometrically at 532 and 600 nm. Absorbance at 600 nm was subtracted from the absorbance at 532 nm (as an index of non-specific turbidity) and the concentration of TBARS was calculated using the Lambert-Beer law with an extinction coefficient εΜ = 155 mM^−1^ cm^−1^ and expressed as malondialdehyde (MDA) equivalents (nmol g^−1^) in line with Du and Bramlage [102].

#### 4.3.8. Osmolytes

Fresh leaf tissue was homogenized in distilled water (1 fresh tissue per 4 mL distilled water). The homogenate was centrifuged at 4000 rpm for 15 min at RT and the recovered supernatant was analyzed. Its osmolarity was determined using an automatic freezing point depression osmometer (Digital Osmometer, Roebling, Berlin, Germany) calibrated with sodium chloride solutions.

#### 4.3.9. Abscisic Acid

Approximately 1 g of leaf tissue was homogenized in a mortar with 3 mL of water, the mixture was centrifuged at 4000 rpm for 15 min at RT and the supernatant was collected and then stored at −80 °C until analysis. The abscisic acid (ABA) concentration was determined by an indirect enzyme linked immuno-sorbent assay based on the use of DBPA1 monoclonal antibody, raised against S(+)-ABA following the procedures of Trivellini et al. [103].

### 4.4. Statistical Analyses

Data obtained from physiological analyses were subjected to a two-way ANOVA, whereas data related to gene expression analysis were subjected to a three-way ANOVA. Differences among means were determined using the Tuckey post-test (*p* < 0.05). Statistics were performed using GraphPad Prism version 6 or 8 for Windows (GraphPad Software, La Jolla California USA, www.graphpad.com). Additional information is reported in each figure’s legend.

## 5. Conclusions

In conclusion, results obtained in this work allowed understanding of some of the mechanisms used by *Diplotaxis tenuifolia* L. to cope with a significant level of salt stress, in response to a foliar treatment with a borage extracts, and the combination of both factors. Moreover, it also allowed us to study the pattern of expression of several transcription factors belonging to different families involved in salt stress responses over time. Transcription factors were found to be involved in the regulation of several pathways such as the biosynthesis of cuticular waxes, phospholipids and brassinosteroids, the sugar metabolism, and the intracellular transport. The results obtained in this work about the response of rocket to the borage extract application as a biostimulant highlighted the difficulty of determining the exact mode of action of these products.

## Figures and Tables

**Table 1:** Phenolic index and anthocyanin concentration in rocket leaves treated with water (CONTROL) and with borage extract (TREATMENT) and subjected to salt stress (200 mM). Measures were taken after one day, two days, and four days of stress. Values are means ± SE (*n* = 4). Data were subjected to two-way ANOVA. Different letters, where present, represent significant differences (*p* < 0.05).

Stress	Treatment	Phenolic Index [ABS_320 nm_ g^−1^]			Anthocyanin [Cyanidin eq. mg/100 g]		
		1 DAS	2 DAS	4 DAS	1 DAS	2 DAS	4 DAS
**NO STRESS**	CONTROL	19.67 ± 2.15	13.83 ± 3.62	20.00 ± 1.88	19.59 ± 1.54	19.31 ± 2.02	21.17 ± 1.71
**NO STRESS**	TREATMENT	19.87 ± 0.86	20.10 ± 0.63	19.06 ± 0.79	21.27 ± 0.58	20.91 ± 0.44	19.43 ± 0.54
**STRESS**	CONTROL	18.22 ± 0.94	17.58 ± 0.86	17.43 ± 1.08	19.45 ± 0.26	19.45 ± 0.63	19.22 ± 0.77
**STRESS**	TREATMENT	18.19 ± 1.84	16.45 ± 1.18	16.37 ± 0.80	18.74 ± 1.67	17.55 ± 1.01	18.00 ± 0.87

## Supplementary Materials

The following are available online at https://www.mdpi.com/2223-7747/9/1/20/s1, Figure S1: Changes in the expression of DtRD29A in rocket leaves treated with water (CONTROL) and with borage extract (TREATMENT) and subjected to salt stress (200 mM). Measures were taken 2, 4, 6, 9, 24 h after the initial exposure to salt stress, Figure S2: Changes in the expression of DtDREB2A (a), DtERF107 (b), DtERF003 (c), DtERF039 (d) in rocket leaves treated with water (CONTROL) and with borage extract (TREATMENT) and subjected to salt stress (200 mM). Measures were taken 2, 4, 6, 9, 24 h after the initial exposure to salt stress, Figure S3: Changes in the expression of DtbHLH122 (a), DtBEE2 (b), DtHBI1-like (c) and DtIBH1-like (d) in rocket leaves treated with water (CONTROL) and with borage extract (TREATMENT) and subjected to salt stress (200 mM). Measures were taken 2, 4, 6, 9, 24 h after the initial exposure to salt stress, Figure S4: Changes in the expression of DtMYB30 (a) and DtMYB94 (b) in rocket leaves treated with water (CONTROL) and with borage extract (TREATMENT) and subjected to salt stress (200 mM). Measures were taken 2, 4, 6, 9, 24 h after the initial exposure to salt stress, Figure S5: Changes in the expression of DtNAC29 (a), DtNAC72 (b), DtANAC69 (c), DtNAC92 (d) and DtNAC019 (e) in rocket leaves treated with water (CONTROL) and with borage extract (TREATMENT) and subjected to salt stress (200 mM). Measures were taken 2, 4, 6, 9, 24 h after the initial exposure to salt stress, Figure S6: Changes in the expression of DtC3H49 (a) and DtZAT12-like (b) in rocket leaves treated with water (CONTROL) and with borage extract (TREATMENT) and subjected to salt stress (200 mM). Measures were taken 2, 4, 6, 9, 24 h after the initial exposure to salt stress, Figure S7: Changes in the expression of DtABF3 (a) and DtbZIP63 (b) in rocket leaves treated with water (CONTROL) and with borage extract (TREATMENT) and subjected to salt stress (200 mM). Measures were taken 2, 4, 6, 9, 24 h after the initial exposure to salt stress, Figure S8: Changes in the expression of DtWRKY54 in rocket leaves treated with water (CONTROL) and with borage extract (TREATMENT) and subjected to salt stress (200 mM). Measures were taken 2, 4, 6, 9, 24 h after the initial exposure to salt stress, Figure S9. Changes in the expression of DtHB12 (a) and DtHB7 (b) in rocket leaves treated with water (CONTROL) and with borage extract (TREATMENT) and subjected to salt stress (200 mM). Measures were taken 2, 4, 6, 9, 24 h after the initial exposure to salt stress, Figure S10: Changes in the expression of DtRABC2B in rocket leaves treated with water (CONTROL) and with borage extract (TREATMENT) and subjected to salt stress (200 mM). Measures were taken 2, 4, 6, 9, 24 h after the initial exposure to salt stress, Figure S11: Changes in the expression of an unknown transcription factors named Unknown2 in rocket leaves treated with water (CONTROL) and with borage extract (TREATMENT) and subjected to salt stress (200 mM). Measures were taken 2, 4, 6, 9, 24 h after the initial exposure to salt stress, Table S1: Statistical results of gene expression analyses on rocket plants treated with borage extract and grown under salt stress condition. “S” means STRESS, “T” means TREATMENT, “t” means TIME, “x” means the INTERACTION between factors, Table S2: Results of ANOVA of physiological analyses on rocket plants treated with borage extract and grown under salt stress condition after 1, 2 and 4 days from the sawing (DAS). “S” means STRESS, “T” means TREATMENT, “x” means the INTERACTION between factors, Table S3: Transcription factors selected from the Diplotaxis tenuifolia RNAseq EST database, grouped by family and divided according to the results obtained by Cavaiuolo et al., (2017), Table S4: Accession number, primers sequences and melting temperature (Tm) for qRT-PCR analysis.
