# Supplementary material for: Transcriptional Regulation in Rocket Leaves as Affected by Salinity
Source: Plants (Basel). 2019 Dec 23;9(1):20. doi: 10.3390/plants9010020 (PMC7020146; doi:10.3390/plants9010020)
Supplement: Supplementary file 1 [file plants-09-00020-s001.pdf]

plants

MDPI

Supplementary material

Table S1. Statistical results of gene expression analyses on rocket plants treated with borage extract and grown under salt stress condition. "S" means STRESS, "T" means TREATMENT, "t" means TIME, "x" means the INTERACTION between factors.

Gene	S	T	t	S x T	S x t	T x t	S x T x t
DtRD29A	**	**	**	**	**	**	**
DtDREB2A	**	ns	**	ns	**	ns	**
DtERF039	**	ns	**	ns	**	*	**
DtERF003	**	**	**	**	ns	ns	*
DtERF107	ns	**	**	ns	**	**	**
DtbHLH122	**	ns	**	ns	**	**	**
DtBEE2	**	ns	*	**	**	*	**
DtHBI1-like	**	ns	**	**	ns	ns	ns
DtIBH1-like	ns	ns	*	*	ns	**	**
DtMYB30	ns	*	**	ns	**	**	**
DtMYB94	**	ns	**	ns	**	*	**
DtNAC019	**	ns	**	ns	**	ns	ns
DtNAC72	**	**	**	**	**	ns	**
DtNAC29	**	**	**	ns	**	ns	ns
DtNAC69	**	ns	**	ns	ns	*	**
DtNAC92	*	*	**	ns	**	**	**
DtC3H49	**	ns	**	ns	*	ns	**
DtZAT12-like	ns	*	**	ns	ns	**	ns
DtbZIP63	**	**	**	**	**	**	**
DtABF3	**	ns	**	ns	**	**	**
DtWRKY54	**	**	**	**	**	**	ns
DtHB12	**	*	**	**	**	**	**
DtHB7	**	ns	**	**	**	ns	ns
DtRABC2B	**	**	**	*	**	**	**
UNKNOWN2	ns	*	**	ns	ns	**	**

* and ** indicate respectively significant differences at $p \leq 0.05$ and $p \leq 0.01$ probability level, ns indicates no significant difference.

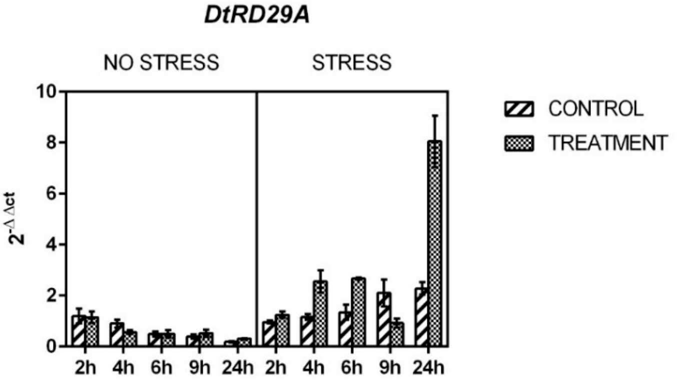

Figure S1. Changes in the expression of *DtRD29A* in rocket leaves treated with water (CONTROL) and with borage extract (TREATMENT) and subjected to salt stress (200 mM). Measures were taken 2, 4, 6, 9, 24 h after the initial exposure to salt stress. Values are means ± SE ($n = 6$). Data were subjected to three-way ANOVA.

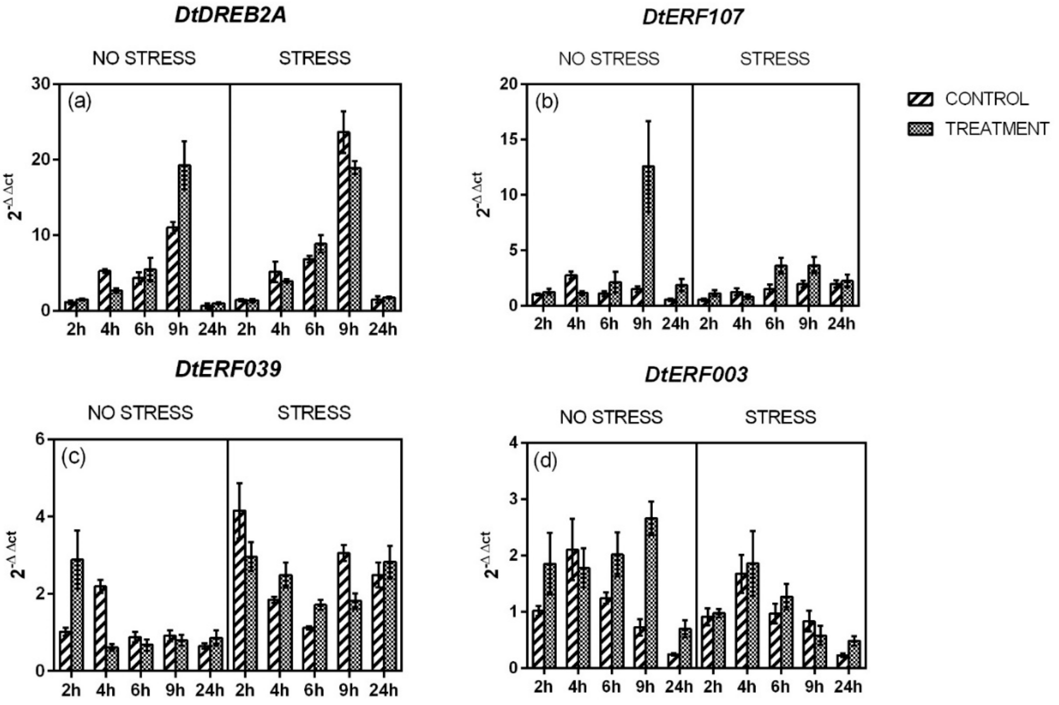

Figure S2. Changes in the expression of *DtDREB2A* **(a)**, *DtERF107* **(b)**, *DtERF003* **(c)**, *DtERF039* **(d)** in rocket leaves treated with water (CONTROL) and with borage extract (TREATMENT) and subjected to salt stress (200 mM). Measures were taken 2, 4, 6, 9, 24 h after the initial exposure to salt stress. Values are means ± SE (*n* = 6). Data were subjected to three-way ANOVA.

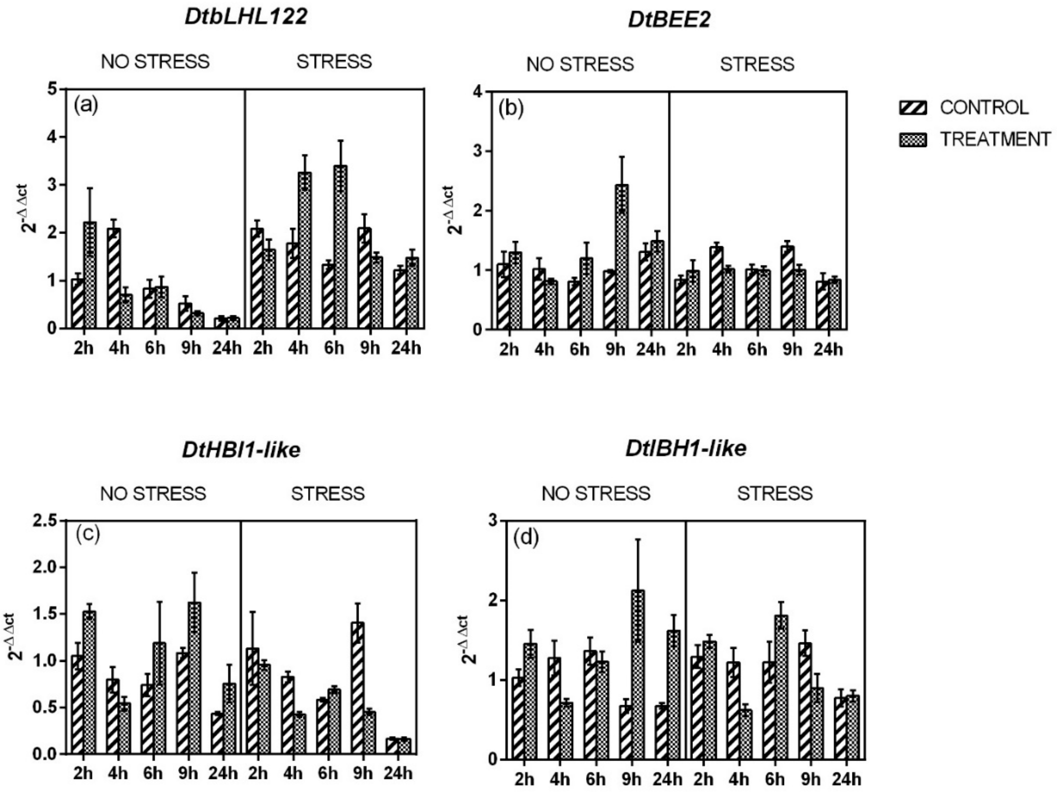

Figure S3. Changes in the expression of *DtbHLH122* **(a)**, *DtBEE2* **(b)**, *DtHBI1-like* **(c)** and *DtIBH1-like* **(d)** in rocket leaves treated with water (CONTROL) and with borage extract (TREATMENT) and

subjected to salt stress (200 mM). Measures were taken 2, 4, 6, 9, 24 h after the initial exposure to salt stress. Values are means ± SE (n = 6). Data were subjected to three-way ANOVA.

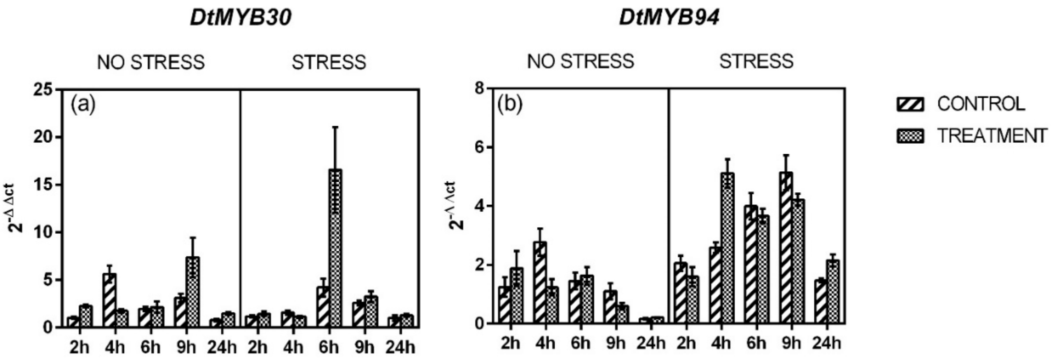

Figure S4. Changes in the expression of *DtMYB30* **(a)** and *DtMYB94* **(b)** in rocket leaves treated with water (CONTROL) and with borage extract (TREATMENT) and subjected to salt stress (200 mM). Measures were taken 2, 4, 6, 9, 24 h after the initial exposure to salt stress. Values are means ± SE (n = 6). Data were subjected to three-way ANOVA.

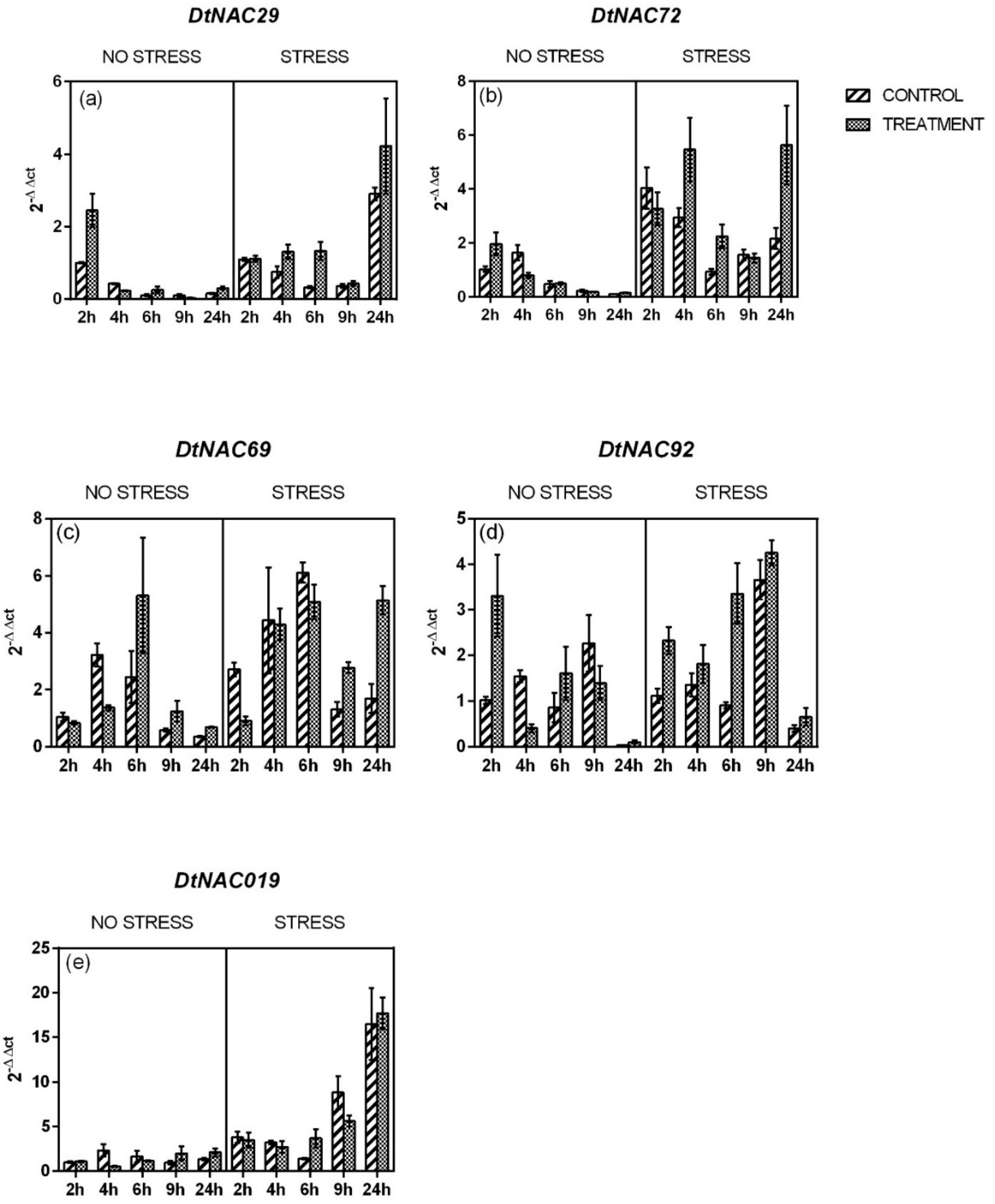

Figure S5. Changes in the expression of *DtNAC29* **(a)**, *DtNAC72* **(b)**, *DtNAC69* **(c)**, *DtNAC92* **(d)** and *DtNAC019* **(e)** in rocket leaves treated with water (CONTROL) and with borage extract (TREATMENT) and subjected to salt stress (200 mM). Measures were taken 2, 4, 6, 9, 24 hours after the initial exposure to salt stress. Values are means ± SE (n = 6). Data were subjected to three-way ANOVA.

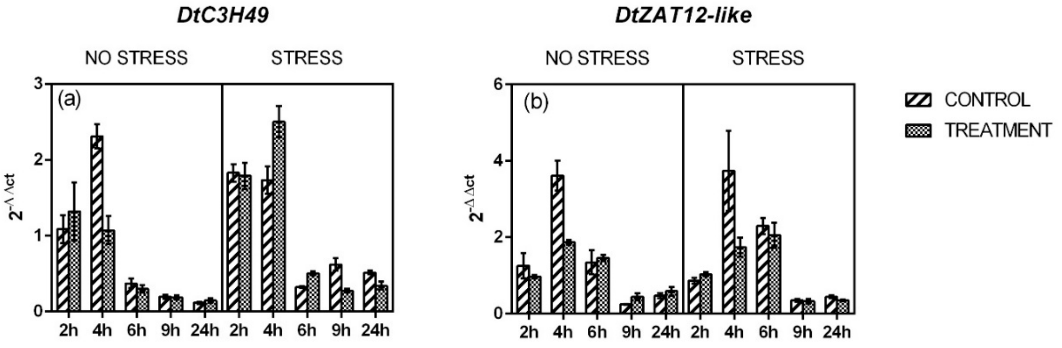

Figure S6. Changes in the expression of DtC3H49 (**a**) and DtZAT12-like (**b**) in rocket leaves treated with water (CONTROL) and with borage extract (TREATMENT) and subjected to salt stress (200 mM). Measures were taken 2, 4, 6, 9, 24 h after the initial exposure to salt stress. Values are means ± SE (n = 6). Data were subjected to three-way ANOVA.

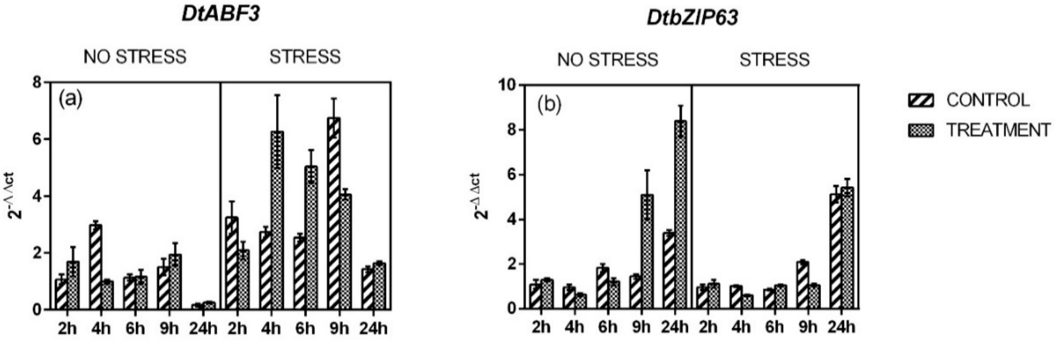

Figure S7. Changes in the expression of *DtABF3* **(a)** and *DtbZIP63* **(b)** in rocket leaves treated with water (CONTROL) and with borage extract (TREATMENT) and subjected to salt stress (200 mM). Measures were taken 2, 4, 6, 9, 24 h after the initial exposure to salt stress. Values are means ± SE (*n* = 6). Data were subjected to three-way ANOVA.

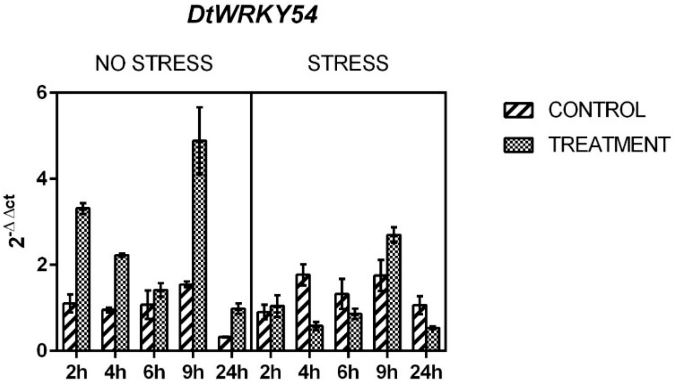

Figure S8. Changes in the expression of *DtWRKY54* in rocket leaves treated with water (CONTROL) and with borage extract (TREATMENT) and subjected to salt stress (200 mM). Measures were taken 2, 4, 6, 9, 24 h after the initial exposure to salt stress. Values are means ± SE (*n* = 6). Data were subjected to three-way ANOVA.

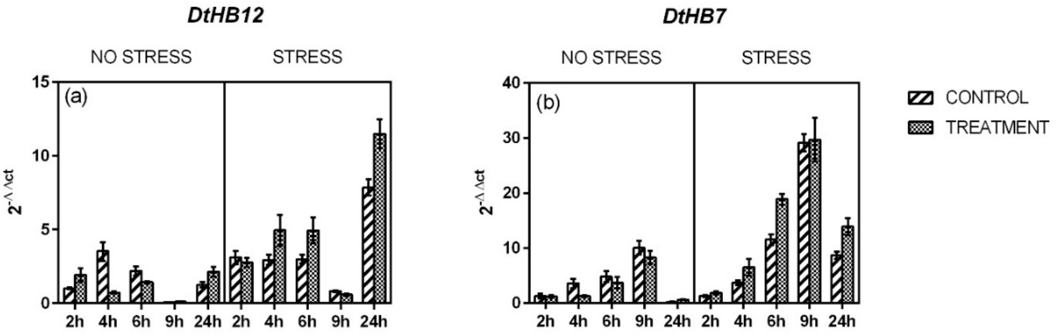

Figure S9. Changes in the expression of *DtHB12* **(a)** and *DtHB7* **(b)** in rocket leaves treated with water (CONTROL) and with borage extract (TREATMENT) and subjected to salt stress (200 mM). Measures were taken 2, 4, 6, 9, 24 h after the initial exposure to salt stress. Values are means ± SE (*n* = 6). Data were subjected to three-way ANOVA.

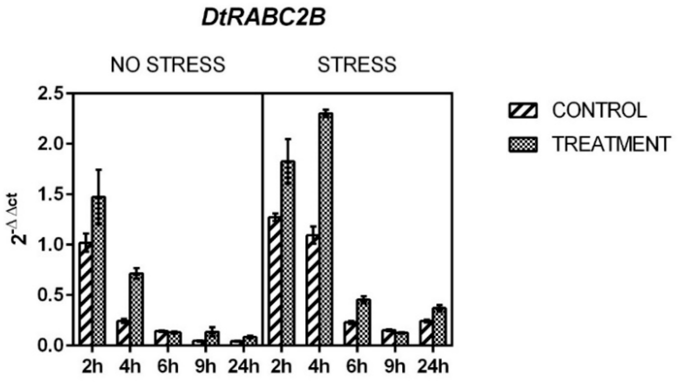

Figure S10. Changes in the expression of *DtRABC2B* in rocket leaves treated with water (CONTROL) and with borage extract (TREATMENT) and subjected to salt stress (200 mM). Measures were taken 2, 4, 6, 9, 24 h after the initial exposure to salt stress. Values are means ± SE (n = 6). Data were subjected to three-way ANOVA.

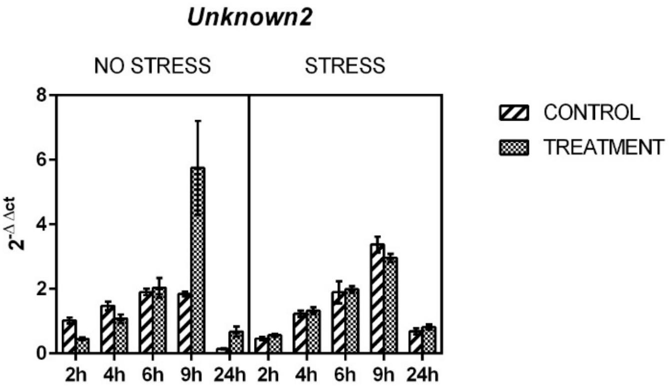

Figure S11. Changes in the expression of an unknown transcription factors named *Unknown2* in rocket leaves treated with water (CONTROL) and with borage extract (TREATMENT) and subjected to salt stress (200 mM). Measures were taken 2, 4, 6, 9, 24 h after the initial exposure to salt stress. Values are means ± SE (n = 6). Data were subjected to three-way ANOVA.

Table S2. Results of ANOVA of physiological analyses on rocket plants treated with borage extract and grown under salt stress condition after 1, 2 and 4 days from the sawing (DAS). "S" means STRESS, "T" means TREATMENT, "x" means the INTERACTION between factors.

	1 DAS			2 DAS			4 DAS		
	T	S	S x T	T	S	S x T	T	S	S x T
chlorophyll	ns	ns	ns	ns	ns	**	**	**	ns
chlorophyll *a* + *b*	ns	ns	ns	ns	ns	ns	ns	*	ns
carotenoids	ns	ns	ns	ns	ns	ns	ns	*	*
phenols	ns	ns	ns	ns	ns	ns	ns	ns	ns
anthocyanin	ns	ns	ns	ns	ns	ns	ns	ns	ns
Fv/Fm	ns	*	*	ns	*	ns	ns	*	ns
PI	ns	*	ns	ns	ns	ns	ns	ns	ns
nitrate	ns	**	ns	ns	**	**	ns	**	ns
reducing sugars	ns	**	ns	ns	ns	ns	ns	ns	ns
total sugars	ns	**	ns	ns	ns	ns	*	ns	ns
lipid peroxidation	ns	**	ns	ns	**	ns	ns	**	ns
osmolytes	ns	ns	ns	ns	ns	ns	ns	ns	ns
abscisic acid	ns	**	ns	*	ns	ns	ns	**	ns

* and ** indicate respectively significant differences at p ≤ 0.05 and p ≤ 0.01 probability level, ns indicates no significant difference.

Table S3. Transcription factors selected from the *Diplotaxis tenuifolia* RNAseq EST database, grouped by family and divided according to the results obtained by Cavaiuolo et al., (2017).

TF Family	Upregulated	Downregulated
**ERF / AP2**	DtERF039	DtDREB2A, DtERF107, DtERF003
**WRKY**		DtWRKY54
**bHLH**	DtbHLH-122	DtBEE2, DtHBI1-like, DtIBH1-like
**ZINC FINGER**	DtC3H49	DtZAT12
**HD-ZIP**	DtHB12, DtHB7	
**b-ZIP**	DtABF3	DtbZIP63
**NAC**	DtNAC72, DtNAC019, DtNAC29, DtNAC69, DtNAC92-NAC59	
**MYB**	DtMYB94	DtMYB30
**-**		UNKNOWN2

Table S4. Accession number, primers sequences and melting temperature (Tm) for qRT-PCR analysis.

Gene	Accession number	Primer Pair	Sequence (5′->3′)	Tm (°C)
DtNAC72	BAJ33621.1	Forward primer	TCATGCACGAGTATCGCCTC	59.97
		Reverse primer	AGAGCTCTGTTCTTCACGGC	60.04
DtHB12	XP_002878419.1	Forward primer	TGGTTTCAGAACAAGAGGGCT	59.51
		Reverse primer	ATTTTCTGGTCCTGTGGTGC	58.38
DtERF039	NP_193408.1	Forward primer	TTAGGATCGGTGCTTGCTGG	60.11
		Reverse primer	CGAACTTTCGTGGGGTCAGA	59.97
DtNAC019	XP_002894409.1	Forward primer	CTGGATACCCAAACCCGACC	60.11
		Reverse primer	ACTCGGGTACAGAACTCGGA	59.96
DtMYB94	XP_002877569.1	Forward primer	ACTGGAGATCCGTGCCTACT	60.03
		Reverse primer	CACCTGTTGCCCAAAAGAGC	59.97
DtbHLH122	XP_002877569.1	Forward primer	AACAGAGGAGACGACGGAGA	59.96
		Reverse primer	GAGCGAGATTATTCGCCGGA	60.04
DtHB7	XP_002882082.1	Forward primer	AGCTGGCTCCACAATGTTCA	59.89
		Reverse primer	AAGTGTGTGAGACGGGACAC	59.90
DtABF3	BAJ34494.1	Forward primer	GACTGCTGAGGAAAGCCACT	59.96
		Reverse primer	GAGGAACTCCGGTGACATCC	59.82
DtC3H49	BAJ33902.1	Forward primer	GTACATGCGGAAATGGTCGC	59.97
		Reverse primer	TCAGAAGACTTCACACCGGC	59.97
DtRABC2B	NP_187602.1	Forward primer	GCTGCTCGTGAGCTGATTTG	59.90
		Reverse primer	ACACGAGCGGTCTTGCTTTA	59.97
DtNAC29	BAJ34610.1	Forward primer	CTTTGTCTGTACCGGTCGCT	60.04
		Reverse primer	ACAAGTTCGACCCATGGCAA	60.18
DtNAC69	NP_192064.1	Forward primer	GACGATTTCGCCAACGACAG	59.91
		Reverse primer	CTCATTTCACACGGCGCATT	59.83
DtNAC92/ NAC59	XP_002875486.1	Forward primer	CGGTCGAACCATCAAAACCG	59.83
		Reverse primer	GCAACCGAGGACAAGGGTTA	59.96
DtDREB2A	AAS58438.1	Forward primer	AGGAAAGTACCCGCGAAAGG	60.04
		Reverse primer	GTCGGAAAGGTACCAAGCCA	59.96
DtBEE2	NP_195372.1	Forward primer	ACTGGTAAAGCCGGTATGCT	59.09

		Reverse primer	CTACGGATCCATGCTGGTGT	59.53
DtbZIP63	XP_002874448.1	Forward primer	TCGCAACTCTCCTCATCGAC	59.55
		Reverse primer	TCCACACTATGCCTCAGGTT	58.34
DtWRKY54	BAJ33964.1	Forward primer	ACTTGGACCGTGGAAGCTAA	58.95
		Reverse primer	ACATCTCAGGGTCTCGCTCA	60.32
DtMYB30	BAJ34042.1	Forward primer	TTCACTTGGCGAAGAAGGCT	59.89
		Reverse primer	CGAGGCATACGTGGTAGAGG	59.69
DtERF107	NP_200967.1	Forward primer	CAGTCGGGCCATGTAGTTGT	60.04
		Reverse primer	GAAACGATGTACCGGAGCCT	59.82
DtIBH1-like	NP_194770.1	Forward primer	TGTCCCCGGTGGAGAGTTTA	60.18
		Reverse primer	ATGCGGTCCTATCGACCAAC	59.90
DtERF003	XP_002874244.1	Forward primer	AGGCAGCAAGGCTAATGTGT	59.96
		Reverse primer	ATTCTTGACGCCGTGAGTGT	59.97
UNKNOWN2	XP_002865887.1	Forward primer	GAGCTTAGCTTCTGAGTGGTGT	60.03
		Reverse primer	ACAACCACCAGCGTAACCAA	60.11
DtHBI1-like	BAJ34477.1	Forward primer	AATGGCTGCAACAGCAACAA	59.54
		Reverse primer	TCCAAAACCAGATCCCGGC	60.00
DtZAT12-like	ADK63406.1	Forward primer	ACTCCGCATAACGGACAAGG	60.11
		Reverse primer	ATTAACTCGACGGTGGAGGC	59.82
DtRD29A	AAB25481.1	Forward primer	TCCACGTGTTGCTTATCCCC	60.04
		Reverse primer	AACTCCGGGATACGGTCAGA	60.03
EF1α	-	Forward primer	TCTTGGTAGACGCCTTCACG	65.3
		Reverse primer	AGGAAGCGGTGTCATTGTTG	65.0
